# Whole-genome sequence and functional annotation dataset of a proteolytic bacterium, *Deinococcus wulumuqiensis* FBCC-B5220

**DOI:** 10.1016/j.dib.2026.112729

**Published:** 2026-03-27

**Authors:** Ahyoung Choi, Jaeduk Goh, Yujin Hwang, Mi-Hwa Lee

**Affiliations:** Biological Resources Research Department, Nakdonggang National Institute of Biological Resources (NNIBR), Sangju 37242, Republic of Korea

**Keywords:** Bacterial genomics, Genome mining, Proteolytic enzymes

## Abstract

This dataset presents the high-quality whole-genome sequence of *Deinococcus wulumuqiensis* FBCC-B5220, a bacterium exhibiting extracellular protease activity isolated from freshwater sediment in Korea. The genome was assembled using a hybrid approach combining Illumina and PacBio platforms, resulting in five contigs totaling 3458,218 bp with a *G* + *C* content of 66.0%. Functional annotation identified 3239 protein-coding sequences, including a specialized repertoire of 45 protease-related genes, such as zinc metalloproteases, ATP-dependent proteases, and serine proteases. Phylogenomic and 16S rRNA gene analyses confirmed its taxonomic identity as *D. wulumuqiensis*, with ANI (97.2%) and dDDH (78.2%) values. The genome sequence and annotation files are available in the NCBI database under the accession number JBSRRC000000000. This dataset provides a high-quality genomic reference for comparative studies within the genus *Deinococcus* and serves as a primary resource for identifying protease-related genes with potential industrial relevance.

Specifications Table

**Table:** 

Subject	Biological Sciences: Genomics
Specific subject area	Microbial genomics and enzyme biotechnology
Type of data	Table (XLSX), Image (TIFF/JPG)
Data collection	Genomic DNA was extracted using DNeasy Blood and Tissue Kit (Qiagen). Sequencing: Illumina NovaSeq 6000 (2 × 151 bp) and PacBio Sequel II (HiFi, 256× coverage). Bioinformatic tools: Flye v2.9, Inspector v1.0.1, Prokka v1.14.6, RAST, InterProScan v5.55, and MEROPS database.
Data source location	Hwasun-gun, Jeollanam-do, Republic of Korea (35° 02′ 54.0″ N, 126° 58′ 23.0″ E).
Data accessibility	Repository name: GenBank/EMBL/DDBJ Data identification number: JBSRRC000000000 Direct URL to data: https://www.ncbi.nlm.nih.gov/nuccore/JBSRRC000000000.1 Instructions for accessing these data: Data is publicly available via the NCBI Nucleotide database.
Related research article	None

## 1 Value of the Data

• This high-quality draft genome serves as a genomic reference for comparative analysis within the genus *Deinococcus*, facilitating strain identification and functional gene mining.
• The correlation of genomic data with phenotypic extracellular protease activity enables the linking of genotype to functional traits for enzyme discovery.
• By providing raw sequencing reads and comprehensive annotation files, this dataset ensures maximum reproducibility and allows benchmarking of assembly pipelines.
• This genome dataset provides a valuable resource for the identification and comparative analysis of protease-related genes and supports future studies investigating their functional and biotechnological relevance.
• The ANI and dDDH results provide genome-based confirmation of strain identity and enable reliable comparative genomic analysis with closely related *Deinococcus* species.

## 2 Background

The genus *Deinococcus* is renowned for its extraordinary resilience to environmental extremes, including ionizing radiation, desiccation, oxidative stress, and various DNA-damaging agents [1]. This polyextremophilic nature is primarily attributed to robust antioxidative defense systems and highly efficient DNA repair mechanisms, positioning *Deinococcus* species as promising microbial reservoirs for discovering enzymes that maintain functionality under harsh industrial conditions [2].

*Deinococcus wulumuqiensis*, first isolated from radiation-polluted soil, is known for its high tolerance to gamma radiation and ultraviolet exposure [3]. Despite its ecological significance, the genomic potential of this species regarding protease production remains largely unexplored. Proteases are among the most versatile and widely utilized industrial enzymes; those derived from extremophiles are particularly valued for their catalytic stability across a broad spectrum of physical and chemical stresses [4,5].

In this study, we report the whole-genome sequence and the comprehensive protease gene repertoire of *D. wulumuqiensis* FBCC-B5220, a strain isolated from freshwater sediment in Korea. The strain demonstrated clear extracellular protease activity via AZCL–casein assays, and subsequent genome annotation revealed a diverse array of protease-related genes.

## 3 Data Description

*Deinococcus wulumuqiensis* strain FBCC-B5220 was isolated from freshwater sediment collected in Hwasun, Republic of Korea. Supplementary Figure S1 shows the image of the agar plate used in an AZCL-casein diffusion assay for the detection of extracellular proteolytic activity. The blue hydrolysis zones around the colonies were measured, showing a mean diameter of 21.50 ± 0.70 mm (*n* = 3).

**Fig. 1:**
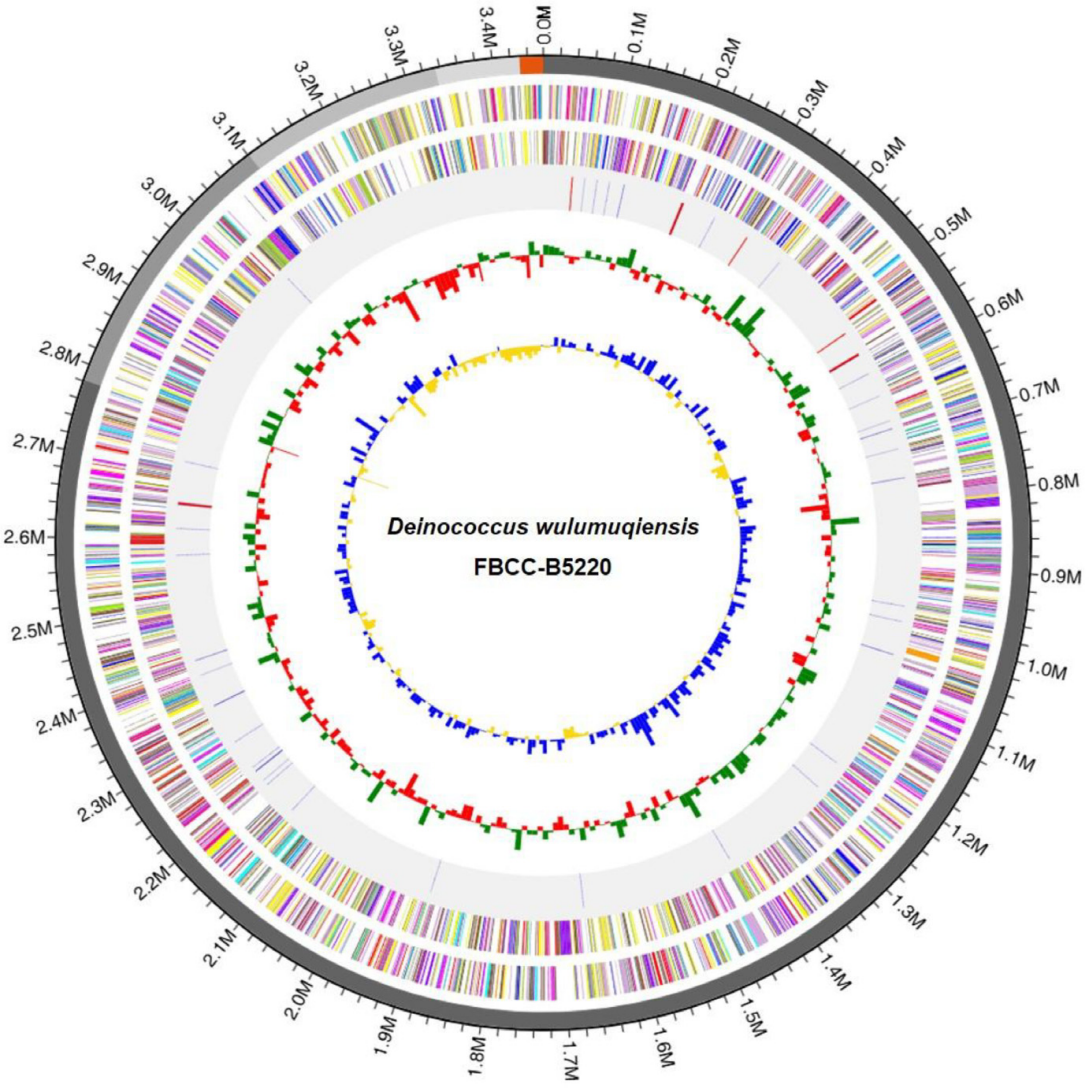
Circular genome map of Deinococcus wulumuqiensis FBCC-B5220. The rings from the outside to the center represent: coding sequences (CDSs) on the forward and reverse strands, RNA genes, G + C content, G + C skew, and protease-related genes (highlighted in red).

Hybrid genome sequencing was performed using the Illumina short-read and PacBio Sequel II long-read platforms. De novo assembly yielded five contigs, with a total genome size of 3458,218 bp, a *G* + *C* content of 66.0%, and an N50 value of 2781,713 bp (Table 1). Genome completeness, assessed using the BUSCO bacteria_odb10 dataset, was 100%. A circular genome map was constructed to visualize the distribution of coding sequences (CDS), *G* + *C* content, and protease-related genes (Fig. 1).

**Table 1:** Genomic statistics and assembly features of *Deinococcus wulumuqiensis* FBCC-B5220.

Feature	Description
Genome size	3458,218 bp
DNA G + C content	66.0%
Assembly status	Draft
Number of contigs	5
N_50_	2781,713 bp
Number of predicted protein-coding genes	3239
rRNA genes (5S, 16S, 23S)	9 (3, 3, 3)
tRNA genes	47
Genome completeness (BUSCO)	100% (bacteria_odb10 dataset)
Sequencing platforms	Illumina NovaSeq 6000 + PacBio Sequel II
Assembly method	Flye v2.9 + Inspector v1.0.1

**Fig. 2:**
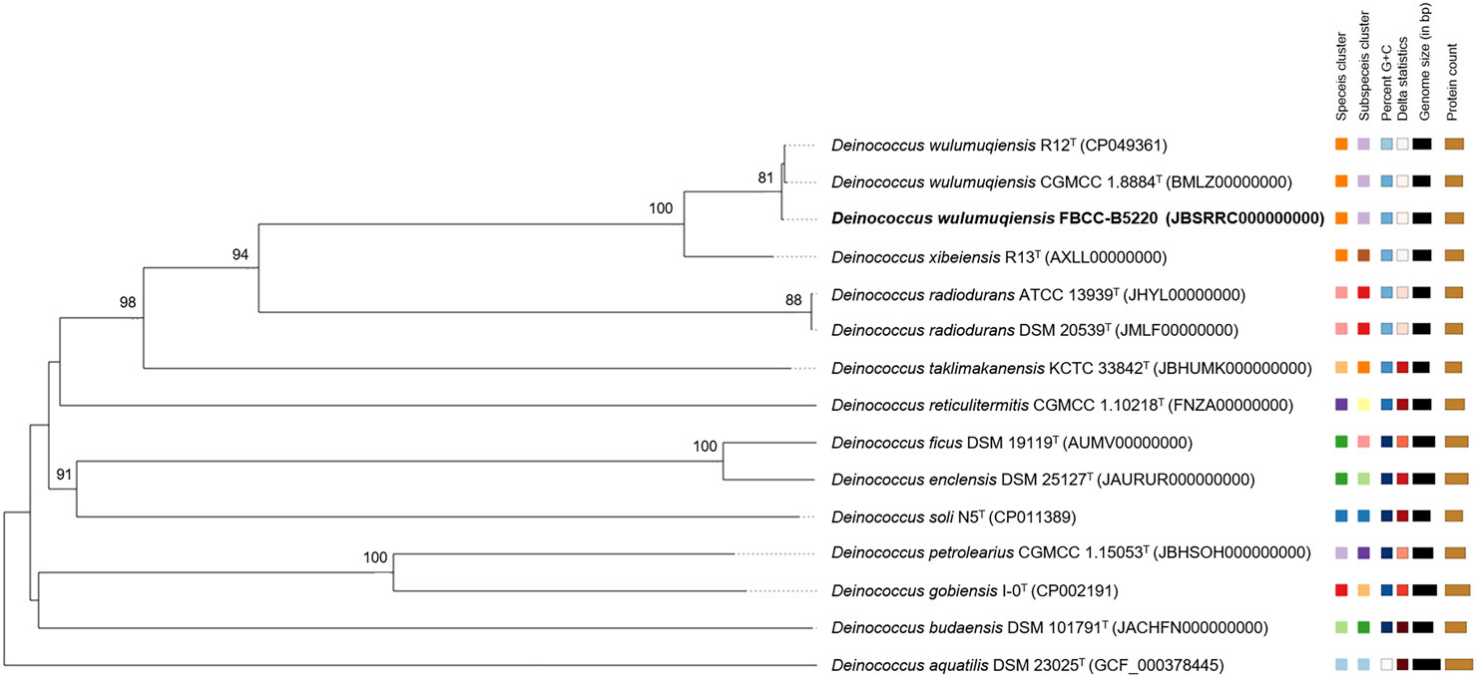
Phylogenomic tree of Deinococcus wulumuqiensis FBCC-B5220 based on Whole-Genome Sequencing (WGS). The tree was constructed using the Genome BLAST Distance Phylogeny (GBDP) algorithm via the Type (Strain) Genome Server (TYGS). Bootstrap values (>70%) from 1000 replicates are shown at the nodes. The tree is midpoint-rooted, with associated genome statistics displayed alongside each strain.

**Fig. 3:**
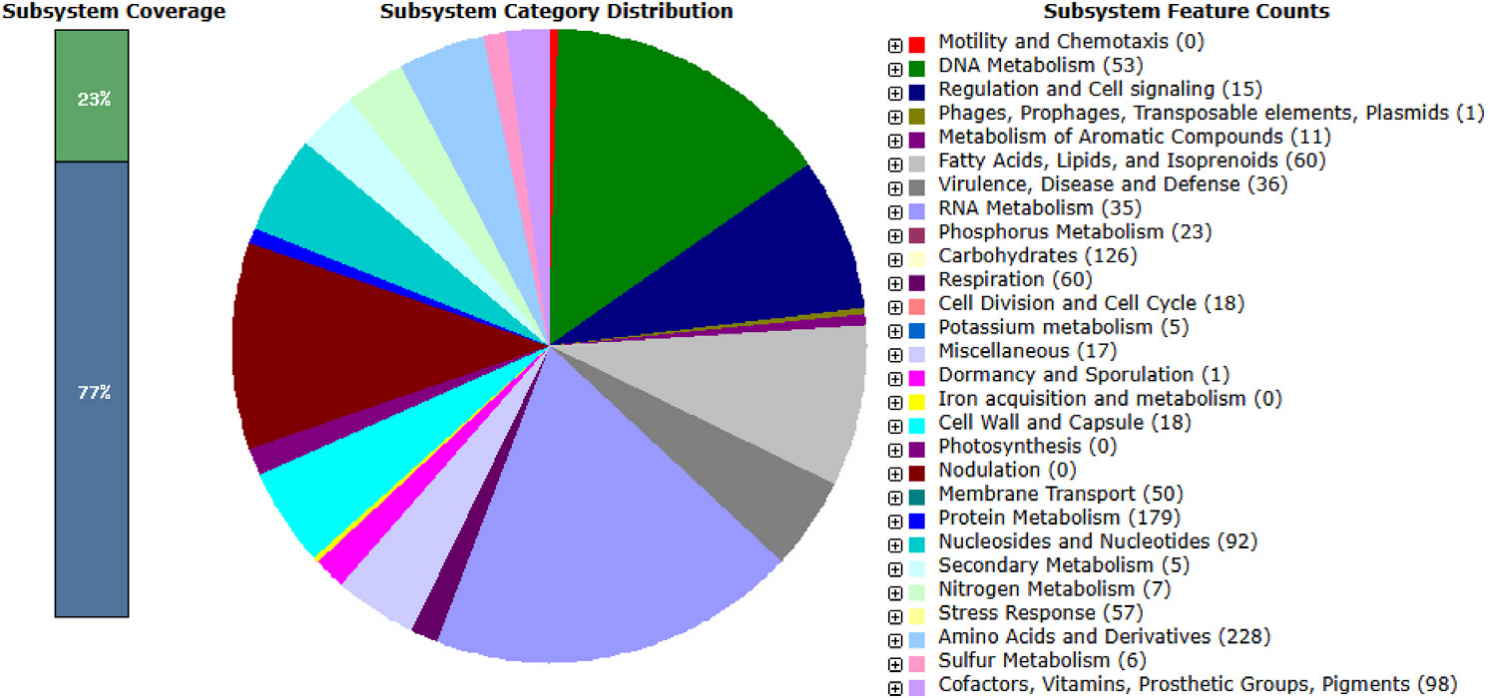
Functional distribution of protein-coding genes in Deinococcus wulumuqiensis FBCC-B5220 based on RAST subsystem categories.

Phylogenomic analysis using Type (Strain) Genome Server (TYGS) and Genome BLAST Distance Phylogeny (GBDP) showed that strain FBCC-B5220 clustered with *Deinococcus wulumuqiensis* R12ᵀ (Fig. 2, Fig. 3). This relationship was also supported by 16S rRNA gene phylogenetic analysis (Supplementary Fig. S2). Pairwise genome comparisons revealed an OrthoANIu value of 97.2% and a digital DNA–DNA hybridization (dDDH) value of 78.2% [[6], [7], [8]].

Table 2 presents the repertoire of 45 protease-related genes categorized by functional classification based on MEROPS, InterProScan, and eggNOG-mapper analysis. These genes consist of zinc metalloproteases (*n* = 8), ATP-dependent proteases (*n* = 7), serine proteases, and uncharacterized proteases (*n* = 19). The complete dataset of these genes, including genomic coordinates, functional predictions, and signal peptide analysis results, is detailed in Supplementary Table S1.

**Table 2:** Functional classification and repertoire of protease-related genes in *Deinococcus wulumuqiensis* FBCC-B5220.

Category	Description	No. of genes
Zinc metalloprotease (FtsH family)	Membrane-bound AAA proteases involved in stress response and protein quality control	3
Subtilase family (serine‑type)	Extracellular serine proteases with broad substrate specificity	6
LexA-type serine protease	DNA damage-inducible self-cleaving repressors; involved in SOS response	3
ATP-dependent protease	Lon-, and La-type intracellular proteases active under stress conditions	8
Other/unspecified proteases	Unclassified or poorly annotated protease domains	25
Total		45

## 4 Experimental Design, Materials and Methods

### 4.1 Strain isolation and cultivation

*Deinococcus wulumuqiensis* strain FBCC-B5220 was isolated from freshwater sediment collected from Hwasuncheon Stream, Hwasun-gun, Jeollanam-do, Republic of Korea (35°02′54.0″ N, 126°58′23.0″ E). The sediment samples were serially diluted in sterile saline (0.85% NaCl (w/v)) and spread onto Reasoner’s 2A (R2A) agar (MBcell, Korea), followed by aerobic incubation at 30 °C for 72 h. A distinct reddish-pigmented colony, indicative of carotenoid-producing *Deinococcus* species, was selected and purified through successive subculturing. The isolate, initially designated as YHS-S-3, was deposited in the Freshwater Bioresources Culture Collection (FBCC) under the accession number FBCC-B5220. For subsequent experiments, the strain was routinely cultivated on R2A agar at 30 °C. For long-term preservation, active cultures were suspended in 20% (v/v) glycerol and maintained at −80 °C.

### 4.2 Extracellular protease activity assay

Extracellular protease activity was assessed using an AZCL–casein agar diffusion assay. Strain FBCC-B5220 was inoculated onto R2A agar supplemented with 0.2% (w/v) AZCL–casein (Megazyme, Ireland) and incubated at 30 °C for 72 h. To facilitate substrate diffusion, an 8 mm sterile paper disc was placed at the center of the inoculation site. Proteolytic activity was evaluated by measuring the diameter of the blue hydrolysis zone resulting from enzymatic cleavage of the chromogenic substrate. Measurements were recorded in millimeters (mm), and assays were performed in triplicate. A non-proteolytic strain (FBCC-B3671) was included as a negative control.

### 4.3 Genome sequencing and assembly

For genomic DNA extraction, *Deinococcus wulumuqiensis* FBCC-B5220 was cultivated on Reasoner’s 2A (R2A) agar (MBcell, Korea) under aerobic conditions at 30 °C for 72 h. Genomic. Genomic DNA was extracted from exponentially growing cultures using the DNeasy Blood and Tissue Kit (Qiagen, USA) following the manufacturer’s instructions. DNA integrity and concentration were rigorously evaluated using a NanoDrop spectrophotometer and a Qubit 4.0 fluorometer (Thermo Fisher Scientific, USA). Whole-genome sequencing was conducted at Theragen Bio (Suwon, Republic of Korea) using a hybrid strategy: Illumina NovaSeq 6000 paired-end reads (2 × 151 bp) provided high base-level accuracy, while PacBio Sequel II HiFi reads (microbial SMRTbell library) ensured structural contiguity. The quality of Illumina and PacBio reads was assessed using FastQC v0.11.9 and NanoPlot v1.41.0, respectively. De novo assembly was performed using Flye v2.9, and the resulting assembly was further polished for consensus accuracy using Inspector v1.0.1. Genome completeness and potential contamination were validated using BUSCO v5.1.3 (bacteria_odb10 dataset) [9] and CheckM v1.2.2. The assembly demonstrated 100% completeness and 0% contamination, confirming the high quality and purity of the genomic data.

### 4.4 Genome annotation and protease gene mining

Genome annotation was performed using multiple independent pipelines for robust cross-validation. Initial gene predictions were generated via Prokka v1.14.6 [10] and the RAST server [11], then refined with the RASTtk v2.0 pipeline. Final structural and functional annotations were obtained through the NCBI Prokaryotic Genome Annotation Pipeline (PGAP) [12] and the EzBioCloud Whole Genome pipeline.

Protease-related genes were identified through comprehensive keyword searches of the annotation outputs and subsequently validated via conserved domain analysis against the MEROPS database [13] and InterProScan v5.55 [14]. To determine secretion potential, signal peptides were predicted using SignalP v6.0. Orthology-based functional classification was performed with eggNOG-mapper v2.1.9, and metabolic pathways were reconstructed using the KEGG Automatic Annotation Server (KAAS) via the bi-directional best hit (BBH) method.

### 4.5 Phylogenetic and comparative genomic analysis

The 16S rRNA gene sequence was extracted from the assembled genome and aligned with those of representative *Deinococcus* species using ClustalW [15]. Phylogenetic reconstruction was performed in MEGA v7.0 [16] using the neighbor-joining (NJ) method. Evolutionary distances were computed via the Kimura 2-parameter (K2P) model, and the statistical reliability of the nodes was estimated through 1000 bootstrap replicates [17].

Average nucleotide identity (ANI) was calculated using the OrthoANIu algorithm via the EzBioCloud server [18], and digital DNA–DNA hybridization (dDDH) values were determined using the Genome-to-Genome Distance Calculator (GGDC) v3.0 [19]. Phylogenomic analysis was performed using the TYGS [20], which calculates intergenomic distances using the GBDP algorithm. The final tree was inferred using FastME under default settings.

## Limitations

The dataset presented in this study is derived from the genome sequencing of a single isolate, *Deinococcus wulumuqiensis* FBCC-B5220. As such, this single genome sequence may not fully represent the complete genetic diversity (pan-genome) or the complex evolutionary dynamics, such as horizontal gene transfer, within the species. While providing a high-quality reference, further sequencing of multiple distinct isolates would be required to achieve a more comprehensive understanding of the species' full genetic potential.

## Ethics Statement

The authors have read and followed the ethical requirements for publication in Data in Brief and confirming that the current work does not involve human subjects, animal experiments, or any data collected from social media platforms.

## CRediT Author Statement

**Ahyoung Choi:** Conceptualization, Data curation, Formal analysis, Investigation, Writing – original draft, Writing – review & editing, Project administration. **Jaeduk Goh:** Supervision, Resources, Project administration, Writing – review & editing. **Yujin Hwang:** Data curation, Formal analysis, Writing – review & editing. **Mi-Hwa Lee:** Supervision, Writing – review & editing.

## Data Availability

The datasets generated during this study are available in the NCBI repository. The genome assembly is accessible under accession number JBSRRC000000000. Associated BioProject, BioSample, and SRA records are available under PRJNA1305641, SAMN50612395, and SRR34986218, respectively.

## Appendix Supplementary materials

Image, application 1
