# Supplementary material for: Whole-genome sequence and functional annotation dataset of a proteolytic bacterium, Deinococcus wulumuqiensis FBCC-B5220
Source: Data Brief. 2026 Mar 27;66:112729. doi: 10.1016/j.dib.2026.112729 (PMC13090655; doi:10.1016/j.dib.2026.112729)
Supplement: Supplementary file 1 [file mmc1.docx]

**Supplementary Materials**

**Article title: Whole-genome sequence and functional annotation dataset of a proteolytic bacterium, *Deinococcus wulumuqiensis* FBCC-B5220**

**Authors: Ahyoung Choi*; Jaeduk Goh, Yujin Hwang, Mi-Hwa Lee**

**Affiliations:** Biological Resources Research Department, Nakdonggang National Institute of Biological Resources (NNIBR), Sangju 37242, Republic of Korea

**Corresponding author’s email address:** [aychoi@nnibr.re.kr](mailto:aychoi@nnibr.re.kr)


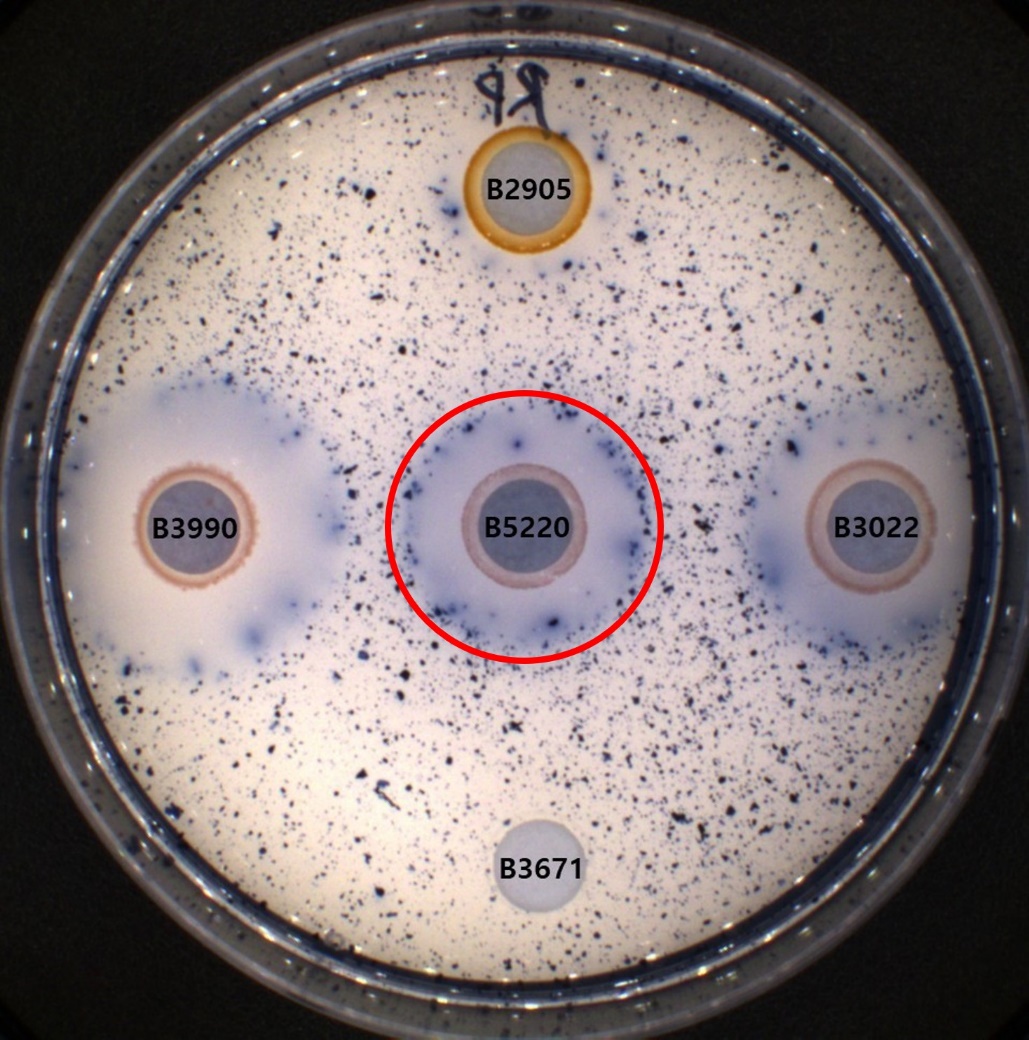


**Fig. S1.** Extracellular protease activity of *Deinococcus wulumuqiensis* strain FBCC-B5220. A clear blue hydrolysis zone on AZCL–casein R2A agar after 72 h of incubation at 30 °C indicates the enzymatic cleavage of the chromogenic substrate by extracellular proteases. The strain analyzed in this study (FBCC-B5220) is indicated by a red circle.

**
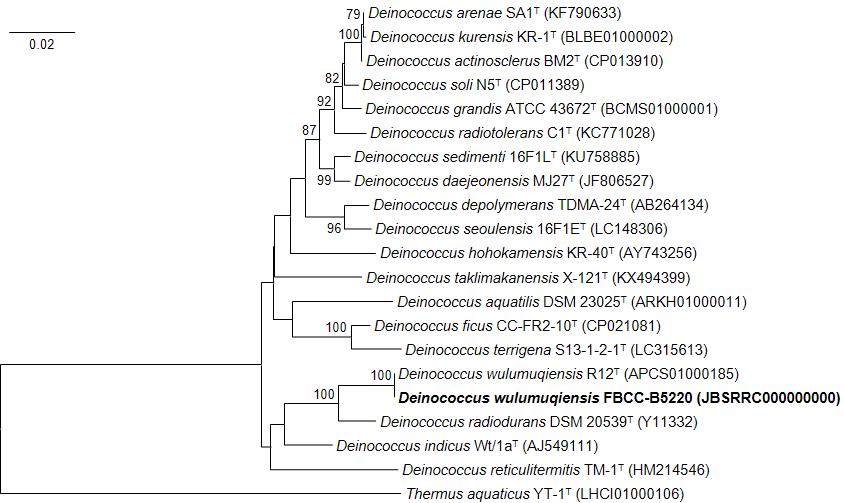
**

**Fig. S2.** Neighbor-joining phylogenetic tree based on 16S rRNA gene sequences showing the phylogenetic position of *Deinococcus wulumuqiensis* strain FBCC-B5220 among representative members of the genus *Deinococcus*. Bootstrap values (>70%) from 1,000 replicates are indicated at the nodes. The strain analyzed in this study is shown in bold. *Thermus aquaticus* YT-1ᵀ (GenBank accession no. LHCI01000106) was used as the outgroup. The scale bar represents 0.02 nucleotide substitutions per site.

**Table S1.** Predicted functional classification and repertoire of protease-related genes in the genome of *Deinococcus wulumuqiensis* FBCC-B5220.

| No. | Feature ID | Type | Start | Stop | Length (bp) | Function |
| --- | --- | --- | --- | --- | --- | --- |
| 1 | fig\|980427.13.peg.211 | CDS | 196529 | 197395 | 867 | YpfJ protein, zinc metalloprotease superfamily |
| 2 | fig\|980427.13.peg.287 | CDS | 277124 | 278401 | 1278 | Zinc protease |
| 3 | fig\|980427.13.peg.288 | CDS | 278398 | 279624 | 1227 | Zinc protease |
| 4 | fig\|980427.13.peg.317 | CDS | 306139 | 306861 | 723 | Zinc metalloprotease |
| 5 | fig\|980427.13.peg.515 | CDS | 491942 | 493528 | 1587 | Alkaline serine exoprotease A precursor (EC 3.4.21.-) |
| 6 | fig\|980427.13.peg.742 | CDS | 710328 | 708472 | 1857 | Cell division-associated, ATP-dependent zinc metalloprotease FtsH |
| 7 | fig\|980427.13.peg.745 | CDS | 712510 | 712836 | 327 | ATP-dependent Clp protease adaptor protein ClpS |
| 8 | fig\|980427.13.peg.749 | CDS | 715026 | 717242 | 2217 | ATP-dependent Clp protease ATP-binding subunit ClpA |
| 9 | fig\|980427.13.peg.784 | CDS | 750436 | 747497 | 2940 | FIG002343: hypothetical protein / FIG001454: Transglutaminase-like enzymes, putative cysteine proteases |
| 10 | fig\|980427.13.peg.892 | CDS | 859734 | 860837 | 1104 | periplasmic serine protease, HtrA/DegQ/DegS family |
| 11 | fig\|980427.13.peg.962 | CDS | 926251 | 924491 | 1761 | Serine protease, subtilase family |
| 12 | fig\|980427.13.peg.991 | CDS | 951611 | 950709 | 903 | Transglutaminase-like enzymes, putative cysteine proteases |
| 13 | fig\|980427.13.peg.1194 | CDS | 1143434 | 1141425 | 2010 | Cell division-associated, ATP-dependent zinc metalloprotease FtsH |
| 14 | fig\|980427.13.peg.1319 | CDS | 1262611 | 1260368 | 2244 | ATP-dependent Clp protease, ATP-binding subunit ClpC |
| 15 | fig\|980427.13.peg.1394 | CDS | 1332514 | 1331942 | 573 | Intracellular protease |
| 16 | fig\|980427.13.peg.1493 | CDS | 1434783 | 1433455 | 1329 | Putative protease |
| 17 | fig\|980427.13.peg.1512 | CDS | 1459289 | 1461205 | 1917 | Serine protease, subtilase family |
| 18 | fig\|980427.13.peg.1759 | CDS | 1688644 | 1689888 | 1245 | carboxyl-terminal protease, putative |
| 19 | fig\|980427.13.peg.1775 | CDS | 1707336 | 1706203 | 1134 | Intramembrane protease RasP/YluC, implicated in cell division based on FtsL cleavage |
| 20 | fig\|980427.13.peg.1803 | CDS | 1736492 | 1738597 | 2106 | Serine protease, subtilase family |
| 21 | fig\|980427.13.peg.1820 | CDS | 1753495 | 1754811 | 1317 | Carboxyl-terminal protease (EC 3.4.21.102) |
| 22 | fig\|980427.13.peg.1864 | CDS | 1797093 | 1799870 | 2778 | FIG015547: peptidase, M16 family / FIG015287: Zinc protease |
| 23 | fig\|980427.13.peg.2066 | CDS | 1997604 | 1998290 | 687 | SOS-response repressor and protease LexA (EC 3.4.21.88) |
| 24 | fig\|980427.13.peg.2166 | CDS | 2098190 | 2096958 | 1233 | Serine protease, subtilase family |
| 25 | fig\|980427.13.peg.2203 | CDS | 2131505 | 2132110 | 606 | ATP-dependent Clp protease proteolytic subunit ClpP (EC 3.4.21.92) |
| 26 | fig\|980427.13.peg.2204 | CDS | 2132107 | 2133318 | 1212 | ATP-dependent Clp protease ATP-binding subunit ClpX |
| 27 | fig\|980427.13.peg.2205 | CDS | 2133452 | 2135896 | 2445 | ATP-dependent protease La (EC 3.4.21.53) Type I |
| 28 | fig\|980427.13.peg.2226 | CDS | 2154418 | 2155242 | 825 | Protein containing transglutaminase-like domain, putative cysteine protease |
| 29 | fig\|980427.13.peg.2350 | CDS | 2268917 | 2270518 | 1602 | Protease IV |
| 30 | fig\|980427.13.peg.2379 | CDS | 2294512 | 2291948 | 2565 | Uncharacterized protease Npun_R3500 |
| 31 | fig\|980427.13.peg.2393 | CDS | 2305127 | 2304654 | 474 | Putative activity regulator of membrane protease YbbK |
| 32 | fig\|980427.13.peg.2495 | CDS | 2390527 | 2391162 | 636 | Uncharacterized protein, similar to the N-terminal domain of Lon protease |
| 33 | fig\|980427.13.peg.2531 | CDS | 2419453 | 2418647 | 807 | Intracellular protease |
| 34 | fig\|980427.13.peg.2627 | CDS | 2509469 | 2509059 | 411 | Mov34/MPN/PAD-1 family protease |
| 35 | fig\|980427.13.peg.2681 | CDS | 2567837 | 2565372 | 2466 | ATP-dependent protease La (EC 3.4.21.53) Type I |
| 36 | fig\|980427.13.peg.2703 | CDS | 2587763 | 2588929 | 1167 | periplasmic serine protease, HtrA/DegQ/DegS family |
| 37 | fig\|980427.13.peg.2877 | CDS | 2754979 | 2753804 | 1176 | Zn-dependent protease with chaperone function |
| 38 | fig\|980427.13.peg.2881 | CDS | 2759679 | 2758984 | 696 | Putative membrane protease YugP |
| 39 | fig\|980427.13.peg.2960 | CDS | 45574 | 43700 | 1875 | Cell division-associated, ATP-dependent zinc metalloprotease FtsH |
| 40 | fig\|980427.13.peg.2963 | CDS | 50714 | 48540 | 2175 | Serine protease, subtilase family |
| 41 | fig\|980427.13.peg.2991 | CDS | 79896 | 82172 | 2277 | Catalase KatE-intracellular protease (EC 1.11.1.6) |
| 42 | fig\|980427.13.peg.3164 | CDS | 250943 | 252358 | 1416 | Serine protease, subtilase family |
| 43 | fig\|980427.13.peg.3200 | CDS | 295554 | 294922 | 633 | SOS-response repressor and protease LexA (EC 3.4.21.88) |
| 44 | fig\|980427.13.peg.3380 | CDS | 145165 | 145851 | 687 | SOS-response repressor and protease LexA (EC 3.4.21.88) |
| 45 | fig\|980427.13.peg.3537 | CDS | 66983 | 66069 | 915 | Membrane protease family protein BA0301 |
